# Conservative treatment of giant spontaneous retropharyngeal hematoma: A case report

**DOI:** 10.1097/MD.0000000000046846

**Published:** 2025-12-26

**Authors:** Guoqiang Du, Yao Bi, Yongtuan Li, Lingling Song, Juan Wang

**Affiliations:** a Department of Otolaryngology Head and Neck Surgery, Qingdao Municipal Hospital, Qingdao, China; b Department of Anesthesiology and Surgery, Qingdao Municipal Hospital, Qingdao, China; c Department of Clinical Laboratory, The Affiliated Hospital of Qingdao University, Qingdao, China.

**Keywords:** absorption time, airway management, SRH, treatment experience

## Abstract

**Rationale:**

Spontaneous retropharyngeal hematoma (SRH) is an occult disease that can cause rapid airway obstruction. Currently, no standardized diagnostic and therapeutic protocols have been established. In this case report, we present the successful conservative management of a patient with a giant SRH, further discuss strategies for airway management and hematoma evacuation, and intend to offer practical insights for the diagnosis and management of SRH.

**Patient concerns:**

A 54-year-old female patient with rapidly progressive neck swelling and respiratory tract obstruction.

**Diagnoses:**

The patient was diagnosed with SRH through computed tomography, magnetic resonance imaging, laryngoscope and ultrasound-guided aspiration biopsy.

**Interventions:**

Timely orotracheal intubation, tracheotomy, and anti-infective therapy.

**Outcomes:**

After these treatments, the patient successfully had the tracheostomy tube removed after 4 weeks and was discharged from the hospital upon recovery.

**Lessons:**

This case emphasizes the importance of early identification of SRH. It also highlights that the treatment focus for SRH lies in airway management and the selection of hematoma evacuation protocols. Furthermore, it underscores the need to consider the regularity of hematoma absorption time in the retropharyngeal space, as well as the timing and approach of surgery that should be taken into account when evacuating the hematoma.

## 1. Introduction

The retropharyngeal space is a narrow space formed anterior to the cervical spine by the visceral and pterygoid fascia, composing primarily of lymph nodes and adipose tissue within a loosely organized stroma, hemorrhagic events in this region are relatively uncommon.^[1]^ Spontaneous retropharyngeal hematoma (SRH) is an even rarer entity, often characterized by obscure etiology and challenging early diagnosis. Once established, the hematoma may expand to compress the airway, leading to life-threatening respiratory obstruction. Clinically, airway management must be prioritized as the primary intervention, with hematoma evacuation serving as the fundamental therapeutic strategy.^[2]^ However, current guidelines for airway intervention and hematoma debridement remain ill-defined, and research on standardized treatment protocols remains scarce. In this report, we present a case of massive SRH involving the posterior mediastinum, dissecting the treatment decision-making process through analysis of the clinical course, observation of disease progression, and synthesis with existing literature.

## 2. Case report

**Figure 1.**
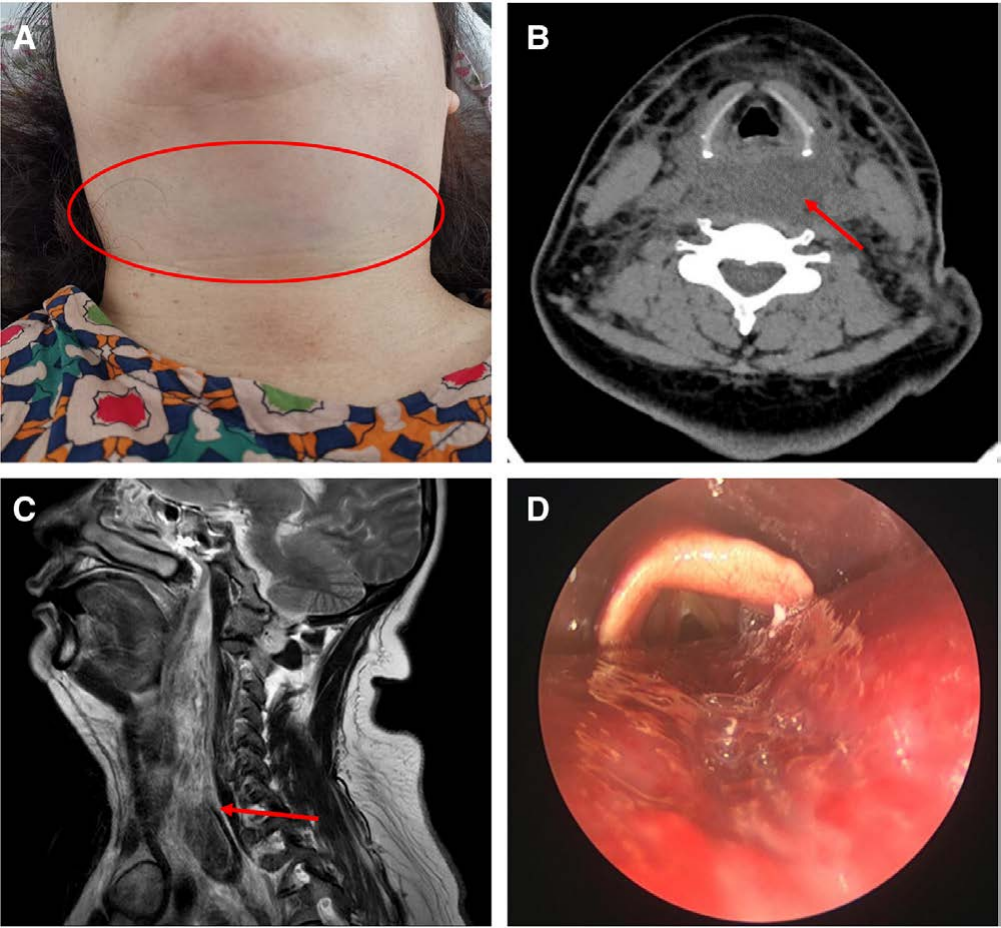
The patient’s physical signs and imaging findings before treatment. (A). Bruised spot on patient’s neck (roundel). (B). Axial CT shows hematoma (arrow) at the level of the arytenoid cartilage. (C). Sagittal MRI shows the extent of the hematoma (arrow). (D). Laryngoscopy shows that the posterior wall of the hypopharynx is bulged, the mucosa shows a dark red color, and it obstructs the posterior three-fourths of the glottic region area. CT = computed tomography, MRI = magnetic resonance imaging.

**Figure 2.**
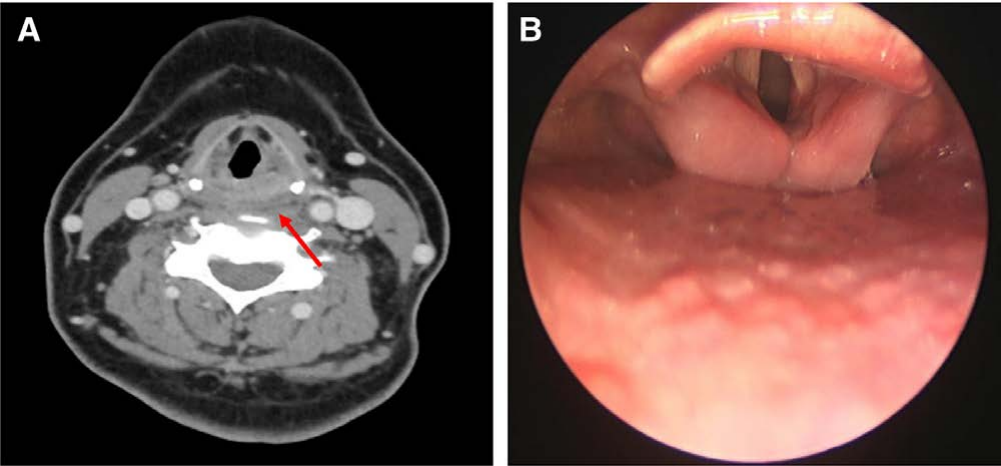
CT scan and laryngoscopic findings after treatment. (A). CT shows hematoma (arrow) resorption at the level of the arytenoid cartilage. (B). The obstruction in the glottic region has disappeared. CT = computed tomography.

A 54-year-old female patient presented to the hospital with neck pain for 4 hours after severe coughing. On physical examination, diffuse swelling was noted in the anterior neck and submental regions, accompanied by scattered subcutaneous ecchymoses. Skin temperature was normal, with no evidence of crepitus or fluctuance (Fig. 1A). Oropharyngeal examination revealed diffuse edema and a violaceous hue of the posterior pharyngeal wall mucosa. Laboratory evaluation showed an elevated C-reactive protein level (32.62 mg/L), while coagulation function tests – including PT, APTT, INR, and factors VIII, IX, XI – were within normal limits, thus ruling out hemorrhagic coagulation disorders. Esophagogastroduodenoscopy demonstrated smooth, normally colored esophageal mucosa. Cervical computed tomography (CT) revealed diffuse iso- and hyperdense shadows extending from the posterior pharyngeal wall to the middle and posterior mediastinum (Fig. 1B). Neck magnetic resonance imaging (MRI) suggested a mesenchymal tissue tumor with superimposed hemorrhage (Fig. 1C). 5 hours after admission, the patient developed inspiratory stridor. Flexible fiberoptic laryngoscopy revealed edematous, dark red mucosa of the posterior hypopharyngeal wall, obscuring the posterior 3-quarters of the vocal folds (Fig. 1D). A differential diagnosis of retropharyngeal space hematoma or abscess was initially entertained. To mitigate the risk of airway compromise, the patient was transferred to the intensive care unit (ICU) for transoral intubation, empirical antibiotic therapy, and preparation for incision and drainage. However, the family declined surgical intervention. On hospital day 3, under ultrasound guidance, a diagnostic needle aspiration was performed from the lateral neck at the cricoid cartilage level, targeting the MRI-suspected tumor site. Approximately 5 mL of purplish-black fluid with small flocculent clots was aspirated, with no evidence of neoplastic tissue, confirming the diagnosis of giant SRH. On day 7, due to poor sputum clearance, a tracheostomy was performed. Four weeks later, repeat neck CT and laryngoscopy demonstrated near-complete resolution of the hematoma (Fig. 2A and B). The tracheostomy tube was decannulated, and the patient was discharged. At 6-month follow-up, no recurrence was noted.

## 3. Discussion

The cause of SRH include strenuous head movements, ruptured neck tumors, coagulation abnormalities, vascular malformation, and obstructive sleep apnea.^[2,3]^ Diagnosis is mainly made by history, physical examination, tests, and imaging. Among them, MRI is important for the diagnosis of SRH, and the acute onset event of hematoma shows high-intensity signal in T1 and T2 weighted sequences.^[4]^ After excluding hemophilia and other coagulation abnormalities as well as vascular malformations, combined with MRI and ultrasound puncture, this case was considered to be caused by vascular rupture and hemorrhage. However, it is regrettable that the patient’s family refused surgical exploration, making it impossible to clarify the etiology. Further follow-up and accumulation of more cases are required for in-depth observational research. Nevertheless, the entire diagnostic and treatment process has provided valuable experience for the conservative management of patients with giant retropharyngeal space hematomas.

Airway protection is a priority category in the treatment of SRH. Early manifestations of SRH are atypical sore throat and neck pain, with further progression causing cervical and thoracic subcutaneous swelling and bruising, anterior displacement of the trachea, and laryngeal and esophageal compression (Capp triad), with varying degrees of upper airway obstruction occurring within 48 hours.^[5]^ Different from what has been reported in the literature, in this case, the patient’s condition progressed rapidly, and airway obstruction occurred within 9 hours. Meanwhile, we also observed that the hematoma was isolated, which indicates that although the tissues in the retropharyngeal space are loose, this space is relatively enclosed. The impact on the surrounding spaces is rather slow, which also leads to the slow absorption of the hematoma in this area and makes it impossible to relieve the compression on the upper respiratory tract in the short term. Tracheal intubation or tracheotomy are the main treatment modalities to keep the airway open.^[6]^ However, precise indications for intubation are lacking. It has been reported that tracheal intubation should be based on the severity of dyspnea and whether the glottis is visible on laryngoscopy, and should also take into account the extent of hematoma expansion, the degree of anterior cervical swelling and subcutaneous bleeding, and the patient’s emotional stability.^[7]^ It has also been suggested that the hematoma diameter on sagittal CT images should be considered as an important reference for early indication of tracheal intubation, and that the range of hematoma diameters for tracheal intubation is 1.5 to 3.6 cm, with a median diameter of 2.5 cm in adults, which is worth taking into account.^[8]^

Currently, there are no guideline recommendations for the management of relatively stable hematomas. Conservative treatment is mainly adopted for most cases. In our opinion, the presence or absence of sustained expansion of the hematoma into other interstitial spaces in the short term is an important factor in the possibility of conservative treatment. For relatively stable retropharyngeal space hematoma, even if the hematoma is extensive and involves the posterior mediastinum, conservative treatment is a better choice under the premise of good airway management.^[7]^ In reviewing the literature, there are no descriptions of the rate of expansion of retropharyngeal hematomas, but it has been reported that hematomas are absorbed within 2 to 4 weeks.^[9]^ We observed the progression of the patient’s hematoma and found that in the early stage of the disease, bleeding rapidly diffused along the retropharyngeal space to form a hematoma. The hematoma enlarged within 3 days and gradually stabilized. It began to shrink on day 6, the mediastinal hematoma was completely absorbed on day 9, and the retropharyngeal space hematoma was basically absorbed on day 28. The complete absorption time of the hematoma was consistent with previous reports, but we fully recorded the hematoma absorption timeline and also confirmed that delayed bleeding is unlikely to occur after bleeding stops. Additionally, blood is an excellent culture medium for bacteria, and any infection in the retropharyngeal space may cause mediastinitis, so active application of antibiotics is required during conservative treatment.

However, the aggressive surgical treatment is still needed if there is persistent active bleeding. Bleeding vessels have been reported to be the superior and inferior thyroid arteries, the superior laryngeal artery, the vessels of the anterior spinal ligament, the internal jugular vein, and the branch vessels of the carotid triangle.^[10]^ It is worth noting that a better surgical field is needed to explore the bleeding point, and the choice of surgical approach is more important as the space of retropharyngeal space is narrow and long which makes it difficult to perform the surgery. Currently, the main surgical approaches include anterior cervical approach, transoral prepharyngeal approach and anterolateral cervical approach. The anterior cervical approach is suitable for the hemorrhage involving the carotid delta, and considering rupture of laryngeal and perithyroidal blood vessels. The transoral approach generally involves a longitudinal incision in the midline of the posterior pharyngeal wall, which can reveal the posterior pharyngeal space from the base of the skull to the level of the epiglottis (C1–C3). The lateral cervical approach is suitable for hemorrhage in the posterior pharyngeal space at the level of C3–C7.^[11]^ For hematomas below the C7 level, it can be removed via anterior superior sternal approach theoretically, which is more traumatic, and has not been reported in the literature. In addition, for the hematoma in the posterior mediastinum, some scholars believe that the mediastinum is lax in tissue, and the hematoma absorbs faster, so it can be left out to avoid mediastinal infection.^[7]^ What’s more, the hematomas due to suspected tumor rupture, the timing of surgery needs to be considered in a comprehensive manner, with some reports suggesting that surgical exploration is recommended 3 months after the acute bleeding event.^[12]^ Overall, for patients whose hematomas still exhibit slow progression after 3 days of conservative treatment, selecting the appropriate surgical approach to remove the hematomas can significantly shorten the patients’ recovery time.

Of course, this case report has certain limitations. Our report is limited to a single case, so we cannot draw comprehensive conclusions regarding the more general progression and prognosis of giant SRH. Additionally, many unknowns remain regarding the differences in treatment strategies for SRH caused by different etiologies. Therefore, it is necessary to conduct summary studies with a larger number of cases.

## 4. Conclusion

This case details the implementation of conservative treatment for giant SRH and documents the sequential hematoma absorption process. It clarifies the roles of laryngoscopy and CT in the early identification of SRH, while underscoring the critical timing for selecting optimal airway management and hematoma evacuation strategies. Collectively, these findings provide clinicians with valuable insights into the diagnosis and management of this rare entity.

## Author contributions

**Conceptualization:** Guoqiang Du.

**Data curation:** Yao Bi.

**Resources:** Yongtuan Li.

**Supervision:** Lingling Song.

**Writing – original draft:** Guoqiang Du.

**Writing – review & editing:** Juan Wang.

**Funding acquisition:** Guoqiang Du, Juan Wang.
